# Prospective study of retention in opioid agonist treatment and contact with emergency healthcare following release from prisons in Victoria, Australia

**DOI:** 10.1136/emermed-2022-212755

**Published:** 2023-02-09

**Authors:** Michael Curtis, Anna L Wilkinson, Paul Dietze, Ashleigh Cara Stewart, Stuart A Kinner, Reece David Cossar, Emily Nehme, Campbell Aitken, Shelley Walker, Tony Butler, Rebecca J Winter, Karen Smith, Mark Stoove

**Affiliations:** 1 Disease Elimination, Burnet Institute, Melbourne, Victoria, Australia; 2 School of Public Health and Preventive Medicine, Monash University, Melbourne, Victoria, Australia; 3 Monash Addiction Research Centre, Monash University, Melbourne, Victoria, Australia; 4 National Drug Research Institute, Curtin University, Melbourne, Victoria, Australia; 5 Victorian Institute of Forensic Medicine, Southbank, Victoria, Australia; 6 Department of Forensic Medicine, Monash University, Melbourne, Victoria, Australia; 7 School of Population Health, Curtin University, Perth, Western Australia, Australia; 8 School of Population and Global Health, University of Melbourne, Melbourne, Victoria, Australia; 9 Centre for Adolescent Health, Murdoch Children’s Research Institute, Melbourne, Victoria, Australia; 10 Griffith Criminology Institute, Griffith University, Brisbane, Queensland, Australia; 11 Research & Evaluation, Ambulance Victoria, Doncaster, Victoria, Australia; 12 Justice Health Research Program, School of Population Health, University of New South Wales, Sydney, New South Wales, Australia; 13 Department of Gastroenterology, St Vincent's Hospital, Melbourne, Victoria, Australia; 14 School of Psychology and Public Health, La Trobe University, Melbourne, Victoria, Australia

**Keywords:** drug overdoses, epidemiology, drug abuse, substance-related disorders

## Abstract

**Background:**

People recently released from prison engage with emergency healthcare at greater rates than the general population. While retention in opioid agonist treatment (OAT) is associated with substantial reductions in the risk of opioid-related mortality postrelease, it is unknown how OAT affects contact with emergency healthcare. In a cohort of men who injected drugs regularly prior to imprisonment, we described rates of contact with ambulance services and EDs, and their associations with use of OAT, in the 3 months after release from prison.

**Methods:**

Self-report data from a prospective observational cohort of men who regularly injected drugs before a period of sentenced imprisonment, recruited between September 2014 and May 2016, were linked to state-wide ambulance and ED records over a 3-month postrelease period in Victoria, Australia. We used generalised linear models to estimate associations between OAT use (none/interrupted/retained) and contact with ambulance and EDs postrelease, adjusted for other covariates.

**Results:**

Among 265 participants, we observed 77 ambulance contacts and 123 ED contacts over a median of 98 days of observation (IQR 87–125 days). Participants who were retained in OAT between prison release and scheduled 3-month postrelease follow-up interviews had lower rates of contact with ambulance (adjusted incidence rate ratio (AIRR) 0.33, 95% CI 0.14 to 0.76) and ED (AIRR 0.43, 95% CI 0.22 to 0.83), compared with participants with no OAT use postrelease. Participants with interrupted OAT use did not differ from those with no OAT use in rates of contact with ambulance or ED.

**Conclusion:**

We found lower rates of contact with emergency healthcare after release among people retained in OAT, but not among people reporting interrupted OAT use, underscoring the benefits of postrelease OAT retention. Strategies to improve accessibility and support OAT retention after leaving prison are important for men who inject drugs.

**WHAT IS ALREADY KNOWN ON THIS TOPIC**

- People recently released from prison have frequent contact with emergency healthcare.
- Opioid agonist treatment (OAT) reduces the risk of drug-related mortality postrelease, but it is unknown how OAT use affects emergency healthcare contact among people recently released from prison.

**WHAT THIS STUDY ADDS**

- This study compares rates of postrelease emergency healthcare contact among men with histories of injecting drug use according to whether they used OAT postrelease.
- Men retained in OAT had lower rates of emergency healthcare contact than men who did not use OAT.
- There was no difference between men who reported interrupted OAT use and no OAT use after release.

**HOW THIS STUDY MIGHT AFFECT RESEARCH, PRACTICE OR POLICY**

- This study highlights that access to and support for retention in OAT is associated with reduced postrelease emergency healthcare use.

## Background

The physical and mental health of people in prison is typically worse than that of the general population.^1^ Imprisonment disrupts healthcare and exacerbates poor health, social and economic circumstances^1 2^; contributing to substantially elevated morbidity and mortality after release.^1^ International^3^ and Australian research^4–9^ has documented high rates of ED and ambulance contact following release from prison, with substance use, mental illness, accidents, injuries and assaults accounting for most postrelease emergency healthcare contacts.^4 5 8 9^

Previous research has sought to understand people at increased risk of postrelease emergency healthcare contact to inform postrelease support. Poor postrelease health-related quality of life,^10^ multiple psychiatric conditions (including dual diagnosis of mental health and substance use disorders),^6 10^ previous inpatient psychiatric admission,^9^ inadequate prerelease discharge planning,^10^ unstable housing^9^ and increased injecting drug use (IDU) frequency^11^ are associated with higher postrelease emergency healthcare contact. Additionally, early postrelease contact with primary healthcare has also been associated with higher hospital admission (inclusive of ED).^12^

Our previous work found people who regularly injected drugs before imprisonment presented to EDs at nearly double the rate of a cohort of Australians released from prison.^8 9^ Retention in opioid agonist treatment (OAT), the frontline treatment for opioid dependence in Australia,^13^ is associated with reduced opioid use,^14 15^ non-fatal^16^ and fatal opioid overdose^17 18^ among people recently released from prison, as well as reduced rates of ED contact among community-recruited cohorts.^19^ Despite this evidence, the impact of retention in OAT on postrelease emergency healthcare contact has received little research attention. Retention in methadone-OAT was associated with reduced ED presentations among >250 000 Canadians with criminal justice system involvement (ie, included people on community-based orders).^20^ A North American study comparing prerelease and postrelease health service contact among people initiating OAT during imprisonment found postrelease rates of ED contact were lower than during the 12 months prior to imprisonment,^21^ but did not account for OAT use after release. Australian studies of postrelease emergency healthcare contact have either omitted OAT use,^4–8 12 22^ or included OAT status at study enrolment as a time-invariant covariate,^9^ thereby omitting transitions on or off of OAT.

Investigation of whether OAT use and retention after release reduces rates of emergency healthcare contact is warranted. Among a cohort of men who regularly injected drugs preceding imprisonment in Victoria, Australia, we describe ambulance and ED contact, and examine differences in rates of emergency healthcare contact in the first 3 months after release from prison by OAT use.

## Methods

### Data sources and participants

We used data from the Prison and Transition Health (PATH) Cohort Study,^23^ a prospective observational study of men (n=400) recruited while imprisoned in Victoria, Australia. Participants were recruited from one minimum-security, one medium-security and one maximum-security prison between September 2014 and May 2016. To be eligible, men were required to be sentenced (not on pretrial detention), aged 18 years or older and self-report at least monthly IDU in the 6 months preceding their recruitment (index) imprisonment episode. Participants completed baseline interviews before release (median 39 days, IQR 15–69) and were invited to participate in three follow-up interviews approximately 3, 12 and 24 months after index release. For consenting participants, survey data were linked with data from administrative health, social service and judicial databases. PATH methodology^23 24^ and cohort characteristics^23 25^ are published elsewhere.

For this study, we used self-report data linked with state-wide administrative ambulance, ED, specialist public mental health service and correctional records; and national primary healthcare (Medicare) and death records. Probabilistic linkage between survey and administrative data was completed by Ambulance Victoria (ambulance), the Centre for Victorian Data Linkage (ED and mental health), Australian Department of Human Services (Medicare) and the Australian Institute of Health and Welfare (death). Victorian Department of Justice and Community Safety (DJCS) completed deterministic data linkage between survey and reimprisonment data.

At the time of manuscript preparation, reincarceration data were unavailable beyond 3-month interviews. Therefore, we only included participants who completed a 3-month follow-up interview in this analysis.

### Patient and public involvement

Participants or the public were not involved in the design, conduct or reporting of this study; however, consultations occurred between researchers and stakeholders including government, support services and peak consumer bodies. Study findings are disseminated to interested participants as requested.

### Outcomes

Our primary outcomes for this study were total counts of contacts with Victorian (1) ambulances and (2) EDs that occurred on or between the dates of index release (determined by DJCS) and 3-month follow-up interview. If participants had multiple ambulance or ED attendances in 1 day, all attendances were counted. However, if multiple ambulance crews attended the same event, only one contact was counted. We also excluded any emergency healthcare contact that occurred from prison, for those reincarcerated during the observation period, determined by usual place of residence recorded in ambulance and ED datasets.

To describe categories of emergency healthcare contacts, we used the International Statistical Classification of Disease and Related Health Problems 10th revision (ICD-10) chapter headings. ED data were supplied with ICD-10 diagnostic codes; if a participant left the ED prior to treatment, no diagnostic code was available. Given frequent misclassification of drug-related ED contacts, we also created ‘drug-related contact’ (ICD-10 codes: F10.0–F19.9, T36.0–T50.9) and ‘opioid-related contact’ (ICD-10 codes: T40.0–T40.4, T40.6, F11.0–F11.9) categories.^26^ Ambulance data do not contain ICD-10 codes, instead containing a ‘final primary assessment’, which describes the paramedic’s assessment of the patient’s main problem at case conclusion, and ‘case nature’, which describes the most likely cause of the main problem. Furthermore, ambulance data do not identify current substance use disorder, required for assignment of drug-related contacts to T or F codes. Therefore, any ambulance contact with a final primary assessment of ‘alcohol’, ‘intoxication’, ‘overdose’ or ‘withdrawal’ were classified as a ‘drug-related contact’, as were contacts with ‘unspecified’, ‘unknown problem’ or ‘other’ recorded for ‘final primary assessment’ where a drug was listed under ‘case nature’. Any ‘drug-related contact’ which included opioids in ‘case nature’ was also assigned to the subgroup ‘opioid-related contact’. We assigned non-drug-related contacts ICD-10 codes using a coding system described previously.^27^

### Covariates

**Figure 1:**
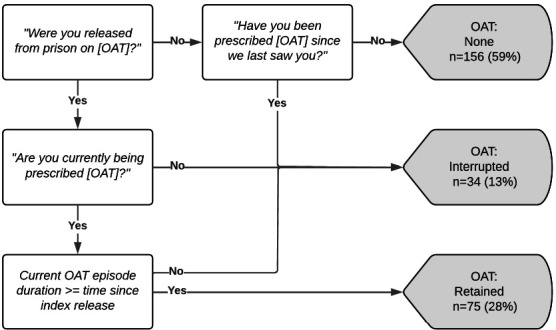
Classification of opioid agonist treatment (OAT) use postrelease using 3-month follow-up survey data (N=265).

Our primary covariate of interest was postrelease OAT use. As Victorian person-level OAT dispensing data are not recorded in any administrative dataset, we used self-report data to classify OAT use postrelease. At 3-month follow-up, we asked participants: *“Since we last saw you, have you been prescribed [OAT (methadone/buprenorphine)]?”*, *“Are you currently [prescribed OAT (methadone/buprenorphine)]?”* and if currently prescribed OAT at interview, *“How long have you been [prescribed OAT (methadone/buprenorphine)] for?”*. Using the responses to these questions, we classified participants into one of three postrelease OAT categories (none/interrupted/retained: figure 1).

We selected additional model covariates a priori following review of health-service utilisation research among people who have been imprisoned^5 9 11 12 20^ and people who inject drugs.^19^ Covariates included age at baseline (years, continuous); Aboriginal and/or Torres Strait Islander (no/yes); ever admitted to a psychiatric facility (pre-index imprisonment, no/yes), determined via linkage to public mental health records; self-reported fair or poor health (vs good, very good or excellent health) at 3-month follow-up (no/yes); self-reported count of times moved accommodation between index release and 3-month follow-up (0/1–2/3+); IDU since baseline interview (no/yes); current psychiatric well-being assessed via the 12-item General Health Questionnaire (GHQ-12,^28^ ordinal, standard GHQ-12 scoring method) and total count of general practitioner consultations (integer) occurring on or between date of index release and 3-month follow-up, determined by linkage to MBS (MBS codes listed in online supplemental appendix 1).

10.1136/emermed-2022-212755.supp1 Supplementary data

### Data analysis

We present descriptive statistics for participants’ characteristics, and assessed for attrition bias by comparing baseline characteristics of participants included and excluded from analyses using independent sample t-tests for normally distributed continuous variables, Mann-Whitney U tests for skewed continuous variables and χ^2^ tests for categorical variables.

To characterise postrelease emergency healthcare contact during the 3 months following prison release, we determined counts of ambulance and ED contacts, grouped by ICD-10 chapter headings, along with drug-related and opioid-related presentations.

To determine associations between counts of emergency healthcare contact and postrelease use of OAT, adjusted for other covariates, we fit two generalised linear models, one with ambulance contacts across 3 months as the outcome and another with ED contacts across 3 months as the outcome. Models used a negative binomial distribution to account for overdispersion of outcome. We considered all participants to be at risk of requiring emergency healthcare, negating the use of zero-inflated models. To account for participants’ differing durations in the community between index release and 3-month follow-up interview, we included the natural logarithm of days spent in the community in each model as an offset term (ie, the rate is defined as count of outcome divided by days spent in the community). We calculated days in the community as days between the date of index release and the date of 3-month follow-up interview, minus the total number of days reimprisoned during this interval. We used likelihood ratio tests to confirm that modelling the number of times participants moved accommodation (0/1–2/3+) as a continuous, rather than categorical variable, did not worsen model fit. We used a complete-case approach to missing data, and reported model estimates as crude (IRR) and adjusted (AIRR) incidence rate ratios with 95% CIs.

We conducted additional analyses to determine whether the inclusion of people who did not use opioids during observation, and were therefore ineligible for OAT, affected associations between OAT use and postrelease emergency healthcare contact. We refit each model described above, restricting the sample to include only participants who reported opioid use (eg, heroin or pharmaceutical opioids, inclusive of prescribed opioid medication) between baseline and 3-month follow-up interview: (1) at least once; (2) on 15 or more days in the 30 days before follow-up interview and (3) on 25 or more days in the 30 days before follow-up interview. We used these thresholds because PATH did not enable determination of opioid use disorder. We used Stata V.14.2^29^ for all analyses.

## Results

### Participant characteristics

Of 400 PATH participants, 5 died before completing a 3-month follow-up interview. Of the remaining 395, 277 (70%) completed a 3-month follow-up interview. We excluded 12 participants from analysis, 9 because linkage to DJCS data occurred before their follow-up interview, preventing calculation of days in the community, and 3 with missing covariate data (psychiatric well-being).

Participants (n=265) contributed 81 person-years at-risk during the study period (median 98 days, IQR 87–125 days). Participants were, on average, 36 years old at baseline; 16% were Aboriginal and/or Torres Strait Islander, 57% moved accommodation at least once since index release and 32% had a historical psychiatric inpatient admission. Among 263 participants for whom data were available, the most common self-reported lifetime mental health diagnoses included depression, anxiety and drug-induced psychosis, reported by 175 (67%), 128 (49%) and 90 (34%) participants, respectively. Between index release and 3-month follow-up, 59% reported no OAT use, 13% reported interrupted OAT use and 28% reported being retained on OAT (table 1). We found no difference between participants included and excluded from analysis on assessed baseline variables (online supplemental appendix 2).

**Table 1:** Characteristics of a cohort of men who regularly injected drugs prior to imprisonment who were released from prison between September 2014 and May 2016 in Victoria, Australia (n=265)

	N (%)
*Baseline*	
Age at baseline (mean (SD))	36 (8)
Aboriginal and/or Torres Strait Islander	43 (16)
Historical psychiatric admission	84 (32)
*Three-month follow-up*	
OAT*	
None	156 (59)
Interrupted	34 (13)
Retained	75 (28)
Fair or poor health	76 (29)
Times moved since index release	
0	113 (43)
1–2	78 (29)
3+	74 (28)
GP visits (median (IQR))	3 (1–6)
Psychiatric well-being (GHQ-12 score; median (IQR))	3 (1–6)
Any IDU since baseline interview	221 (83)

*Versus good, very good or excellent health.

GHQ-12, 12-item General Health Questionnaire; GP, general practitioner; IDU, injecting drug use; OAT, opioid agonist treatment.

### Use of postrelease emergency healthcare services

Seventy-six participants (29%) had at least one contact with emergency healthcare during observation; 46 participants contributed 77 ambulance contacts and 64 participants contributed 123 ED contacts between index release and 3-month follow-up interview. Thirty-four participants had at least one contact with both ambulance and ED. Drug-related contacts (42%) and symptoms, signs and abnormal clinical and laboratory findings, not elsewhere classified (26%) were the most common reasons for ambulance contact. Injury, poisoning and certain other consequences of external causes (31%), and mental and behavioural disorders (24%), were the most common reasons for ED contact (table 2).

**Table 2:** Counts and proportions of most frequent diagnostic categories for ambulance and ED contacts postrelease among a cohort of men (n=265) who regularly injected drugs prior to imprisonment who were released from prison between September 2014 and May 2016 in Victoria, Australia

	Ambulance contacts	ED contacts
	(n=77*)	(n=123†)
ICD-10 chapters		
Injury, poisoning and certain other consequences of external causes	<5‡	38 (31)
Symptoms, signs and abnormal clinical and laboratory findings, not elsewhere classified	20 (26)	18 (15)
Mental and behavioural disorders	6 (8)§	30 (24)
Factors influencing health status and contact with health services	<5	10 (8)
Other ICD-10 chapters	7 (9)	21 (17)
No problem specified	7 (9)	–
Left against advice	–	6 (5)
Drug-related contacts	32 (42)	18 (15)¶
Opioid-related contacts**	17 (22)	6 (5)††

*Total includes drug-related contacts.

†Total excludes drug-related contacts.

‡Excludes drug-related codes: T36.0–T50.9.

§Excludes drug-related codes: F10.0–F19.9.

¶ICD-10 codes: F10.0–F19.9, T36.0–T50.9.

**Subset of drug-related contacts.

††ICD-10 codes: T40.0–T40.4, T40.6, F11.0–F11.9.

ICD-10, International Statistical Classification of Disease and Related Health Problems 10th revision.

### Associations between OAT and postrelease emergency healthcare contact

In adjusted model, we found men retained in OAT between index release and 3-month follow-up had lower rates of ambulance (AIRR 0.33, 95% CI 0.14 to 0.76) and ED (AIRR 0.43, 95% CI 0.22 to 0.83) contact than men who reported no use of OAT postrelease (table 3). We found no difference between rates of emergency healthcare use for men reporting interrupted versus no OAT use. Complete adjusted estimates for each emergency healthcare model are supplied (online supplemental appendix 3).

**Table 3:** Counts of ambulance and ED contacts, mean and SD stratified by postrelease opioid agonist treatment exposure (OAT; none: n=156, interrupted: n=34, retained: n=75), unadjusted (IRR) and adjusted incidence rate ratios (AIRR) and 95% CIs comparing use of emergency healthcare postrelease according to OAT use in the first 3 months postrelease among a cohort of men who regularly injected drugs prior to imprisonment (n=265)

Outcome	Count of contacts	Mean of contacts (SD)	IRR (95% CI)	P value	AIRR* (95% CI)	P value
1. Ambulance contacts	77	0.29 (0.80)				
OAT: none	51	0.33 (0.80)	1.00		1.00	
OAT: interrupted	11	0.32 (0.88)	0.97 (0.45 to 2.09)	0.943	0.54 (0.21 to 1.34)	0.182
OAT: retained	15	0.20 (0.77)	0.66 (0.35 to 1.27)	0.216	0.33 (0.14 to 0.76)	0.009
2. ED contacts	127	0.46 (1.12)				
OAT: none	81	0.52 (1.12)	1.00		1.00	
OAT: interrupted	14	0.41 (1.08)	0.83 (0.42 to 1.67)	0.604	0.54 (0.23 to 1.24)	0.144
OAT: retained	28	0.37 (1.15)	0.77 (0.46 to 1.30)	0.335	0.43 (0.22 to 0.83)	0.012

*Adjusted for age at baseline, Aboriginal and/or Torres Strait Islander, historical psychiatric admission, self-reported fair or poor health, times moved accommodation, count of general practitioner consultations, psychiatric well-being (GHQ-12 score) and any IDU since baseline interview.

GHQ-12, 12-item General Health Questionnaire; IDU, injecting drug use.

Most (77%, 203/265) participants reported at least one instance of opioid use since baseline interview, and were included in secondary analysis. Associations between use of OAT and rates of ambulance and ED contact were consistent with the primary analyses (table 4).

**Table 4:** Secondary analyses of counts of ambulance and ED contacts, mean and SD, unadjusted (IRR) and adjusted incidence rate ratios (AIRR) and 95% CIs comparing use of emergency healthcare postrelease according to opioid agonist treatment (OAT) use in the first 3 months postrelease among a cohort of men who regularly injected drugs prior to imprisonment stratified by postrelease OAT exposure and frequency of opioid use

Outcome	Count of contacts	Mean of contacts (SD)	IRR (95% CI)	P value	AIRR* (95% CI)	P value
Any postrelease opioid† use (n=203)						
*1. Ambulance contacts*	61	0.30 (0.80)				
OAT: none	35	0.37 (0.79)	1.00		1.00	
OAT: partial	11	0.32 (0.88)	0.88 (0.39 to 1.96)	0.751	0.57 (0.21 to 1.49)	0.248
OAT: retained	15	0.20 (0.77)	0.60 (0.30 to 1.19)	0.146	0.34 (0.14 to 0.83)	0.018
*2. ED contacts*	104	0.51 (1.19)				
OAT: none	62	0.66 (1.25)	1.00		1.00	
OAT: partial	14	0.41 (1.08)	0.67 (0.33 to 1.37)	0.273	0.45 (0.19 to 1.09)	0.078
OAT: retained	28	0.37 (1.15)	0.62 (0.36 to 1.08)	0.092	0.37 (0.18 to 0.76)	0.006
15 or more days of opioid† use in the 30 days before follow-up (n=139)						
*1. Ambulance contacts*	44	0.32 (0.84)				
OAT: none	18	0.50 (0.85)	1.00		1.00	
OAT: partial	11	0.39 (0.96)	0.79 (0.32 to 1.99)	0.621	0.81 (0.26 to 2.58)	0.724
OAT: retained	15	0.20 (0.77)	0.44 (0.20 to 0.98)	0.045	0.34 (0.12 to 0.98)	0.045
*2. ED contacts‡*	65	0.47 (1.13)				
OAT: none	24	0.67 (1.07)	1.00		1.00	
OAT: partial	13	0.46 (1.17)	0.73 (0.31 to 1.72)	0.468	0.56 (0.19 to 1.67)	0.300
OAT: retained	28	0.37 (1.15)	0.59 (0.29 to 1.17)	0.128	0.39 (0.16 to 0.92)	0.032
25 or more days of opioid† use in the month before follow-up (n=129)						
*1. Ambulance contacts*	39	0.30 (0.84)				
OAT: none	11	0.44 (1.00)	1.00		1.00	
OAT: partial	11	0.44 (1.00)	1.00 (0.37 to 2.68)	0.995	1.34 (0.39 to 4.69)	0.642
OAT: retained	15	0.20 (0.77)	0.48 (0.20 to 1.16)	0.102	0.41 (0.13 to 1.30)	0.129
*2. ED contacts‡*	60	0.47 (1.17)				
OAT: none	20	0.69 (1.17)	1.00		1.00	
OAT: partial	12	0.48 (1.23)	0.73 (0.29 to 1.82)	0.499	0.59 (0.18 to 1.93)	0.383
OAT: retained	28	0.37 (1.15)	0.56 (0.27 to 1.17)	0.122	0.38 (0.15 to 0.95)	0.039

*Adjusted for age at baseline, Aboriginal and/or Torres Strait Islander, historical psychiatric admission, self-reported fair or poor health, times moved accommodation, count of general practitioner consultations, psychiatric well-being (GHQ-12 score) and any IDU since baseline interview.

†Opioids include heroin as well as licit and illicit pharmaceutical opioids including methadone, buprenorphine, morphine and oxycodone.

‡Aboriginal and/or Torres Strait Islander omitted from analysis as no Aboriginal and/or Torres Strait Islander participant attended ED and used opioids at least 15 days in the month before follow-up interview.

GHQ-12, 12-item General Health Questionnaire; IDU, injecting drug use.

## Discussion

In a cohort of men who injected drugs regularly in the 6 months prior to imprisonment, approximately one in four had contact with emergency healthcare in the 3 months after release from prison. The rate of contact with emergency healthcare among men retained in OAT postrelease was less than half that of men who did not receive OAT during this time.

Our results suggest that, among people released from prison, rates of emergency healthcare contact are elevated among those who regularly injected drugs before imprisonment. For example, the proportion of participants in our study who had contact with an ED during a median of 96 days of observation was approximately three times higher than that reported in a retrospective case-control study of people released from prisons in Canada.^3^ The proportion with ED contact in our study is also greater than reported in studies of people released from prisons in Queensland^12^ and Western Australia,^4^ during 9-month and 12-month observation periods, respectively. Our findings reinforce existing evidence of the elevated risk of adverse health outcomes and increased use of emergency healthcare among people with histories of IDU after release from prison. Effective interventions to reduce occurrence of preventable health events requiring emergency healthcare among people recently released from prison, including programmes supporting access to and retention in OAT, are needed.

Our finding that retention in OAT was associated with lower rates of emergency healthcare contact than no OAT use is consistent with previous findings from community-recruited OAT cohorts^19^ and among people involved in the criminal justice system.^20 21^ For example, a population-level retrospective cohort study of people with a judicial record in British Columbia found that retention in methadone-OAT halved the rate of contact with EDs.^20^ Similarly, an American study of people who initiated OAT during imprisonment found reduced rates of postrelease ED contact compared with the 12 months preceding imprisonment.^21^ While not examined in our study, less frequent emergency healthcare contact among people retained in OAT may relate to reduced opioid-related harms. The weeks following release from prison are associated with higher risk of opioid overdose, due in part to loss of opioid tolerance during imprisonment.^1^ Retention in OAT maintains opioid tolerance at levels that reduce the demand for and effects of illicit opioids.^15 30^ Accordingly, the risk of experiencing drug-related health events necessitating emergency healthcare contact, including opioid overdose,^31^ injecting-related injuries^11^ and infections^32^ and drug-related violence,^33^ is lower among people retained in OAT. However, opioid-related contacts accounted for few of the observed ED contacts, suggesting that OAT retention has benefits not directly related to reduced opioid consumption. Future research with a larger sample size should examine associations between OAT retention and types of emergency healthcare among people recently released from prison.

We found no difference between rates of postrelease emergency healthcare contact for people who did not use OAT and people who reported interrupted OAT use, although our study was likely underpowered to detect this. A previous community-based study of people enrolling in buprenorphine-OAT for the first time found that the proportion of people with at least one opioid-related ED presentation reduced in an approximate dose-response relationship as the proportion of observed days spent on OAT increased.^34^ Furthermore, a recent systematic review and meta-analysis found an increased risk of drug-related mortality during OAT initiation and following OAT discontinuation compared with time spent in treatment, suggesting transitioning on and off of OAT is associated with increased risk of acute health events such as opioid overdose.^35^ Our findings are consistent with this research, suggesting that it is insufficient to make OAT available to people in and transitioning out of prison. OAT programmes for this population must also improve access to treatment and support retention. There are various opportunities to improve OAT retention after prison release, including provision of targeted prerelease and postrelease support to people at increased risk of discontinuation, such as Aboriginal and Torres Strait Islander peoples^36^ and people who initiate OAT in prison,^37^ and reducing the costs associated with OAT.^38^

However, improved access to OAT does not address the broader social and structural determinants of poor health and drivers of high rates of acute health episodes among people recently released from prison. Indeed, improving prerelease discharge planning and transitional healthcare,^9 10^ provision of secure and affordable accommodation^6 9^ and improved access to other harm reduction measures such as take home naloxone^39^ will also likely reduce occurrence of acute health events following release from prison. Furthermore, OAT is a medical treatment, and while its benefits supporting reduced postrelease opioid use,^14 15^ non-fatal^16^ and fatal opioid overdose^17 18^ are well-established, efforts to support people with drug use histories recently released from prison must also focus on empowerment and ensuring that individual agency is not compromised when offering OAT, as has been shown in other settings.^40^ It is also important to ensure that people who choose not to enrol in these programmes are not stigmatised; other forms of support (eg, overdose awareness education) are needed to minimise postrelease risks for these people.

## Strengths and limitations

The combination of linked self-report and administrative data is a strength of our study. Linked administrative ambulance and ED data provided objective measures of emergency healthcare contact; self-report data enabled adjustment for covariates not captured in administrative data. However, our findings have limitations. A key limitation is the inability to determine current opioid use disorder from PATH data. The inclusion of people who did not use opioids or who were not opioid dependent, and therefore were not suitable for OAT, could have impacted results. However, results of secondary analyses restricting the sample to various levels of postrelease opioid use were broadly consistent with the primary analysis. Future research should re-examine this research question among people diagnosed with opioid use disorder. Participants were sentenced men; our results do not generalise to women, minors or people on pretrial detention. Our research took place in Victoria, Australia; differing prison-based and community-based health systems may limit generalisability elsewhere. Future work should seek to include groups excluded from our analysis, and occur across multiple jurisdictions. Reliance on self-report data for OAT exposure classification potentially introduced recall and misclassification bias, and precluded determination of timing of emergency healthcare contacts relative to OAT status among the interrupted OAT group, crucial to improving understanding of the relationship between interrupted OAT use and postrelease emergency healthcare contact. Although we found no differences between participants included and excluded from analysis on select covariates, we cannot exclude attrition bias. Sample size precluded examination of whether associations differed according to OAT medicine. Finally, while receiving OAT is associated with improved outcomes among people with opioid dependence,^19–21 30 32 35^ effective pharmacotherapy for methamphetamine dependence is non-existent.^41^ Methamphetamine is the most commonly used drug among people entering Australian prisons,^2 23^ and is associated with increased emergency healthcare contact.^42^ Initiatives that reduce postrelease methamphetamine-related harms are needed.

## Conclusion

People recently released from prison have frequent contact with emergency healthcare. In men who regularly injected drugs before imprisonment in Victoria, Australia, we found that retention in OAT was associated with reduced rates of contact with ambulance and EDs in the 3 months after release. Our findings highlight the importance of programmes that provide access to OAT and support retention after release from prison. Such programmes are likely to reduce morbidity and mortality outcomes among people experiencing opioid dependence recently released from prison, while also reducing the burden on emergency healthcare.

## Data Availability

Data may be available on request to Victorian Department of Justice and Community Safety ethics approval.
