# Supplementary material for: Prospective study of retention in opioid agonist treatment and contact with emergency healthcare following release from prisons in Victoria, Australia
Source: Emerg Med J. 2023 Feb 9;40(5):347–54. doi: 10.1136/emermed-2022-212755 (PMC10176422; doi:10.1136/emermed-2022-212755)
Supplement: Supplementary data [file emermed-2022-212755supp001.pdf]

Appendix 1. Medicare Benefits Schedule (MBS) codes used to determine count of general practitioner (GP) contacts between date of index release and three-month follow-up interview.

**GP consultation type**	**MBS Item Codes**
Standard Consultation	3, 4, 23, 24
Extended Consultation	36, 37, 44, 47
Mental Health Consultation	2700, 2701, 2712, 2713, 2715, 2717, 2721, 2725
After Hours Consultation	5000, 5020, 5023, 5028, 5040, 5043, 5060

Appendix 2. Baseline participant characteristics of a cohort of men who regularly injected drugs prior to imprisonment who were released from prison between September 2014 and May 2016 in Victoria, Australia, stratified by analysis inclusion

	**Excluded** [a] (N=135) n (%)	**Included** (N=265) n (%)	**p-value**
Age (mean, [SD [b]])	36 (9)	36 (8)	0.424 [c]
Aboriginal and/or Torres Strait Islander	23 (17)	43 (16)	0.836 [d]
Born in Australia	119 (88)	239 (90)	0.529 [d]
Unemployed prior to index imprisonment	122 (90)	230 (87)	0.213 [d]
Completed High school	18 (13)	52 (20)	0.117 [d]
Historical psychiatric admission	36 (27)	84 (32)	0.299 [d]
Self-reported fair or poor health at baseline	35 (26)	60 (23)	0.465 [d]
Times moved in the 12 months before index imprisonment (n=392)			
0	51 (39)	104 (40)	
1–2	28 (21)	70 (27)	0.278 [d]
3+	53 (40)	85 (33)	
C-GHQ-12 [e] Score (median, [IQR [f]])	4 (2–7)	5 (2–7)	0.692 [g]
Days IDU in the month before index imprisonment (median, [IQR [f]])	28 (17–28)	28 (9–28)	0.108 [g]
Receiving OAT at baseline interview	62 (46)	103 (39)	0.175 [d]

[a] Reasons for exclusion: died before three-month follow-up (n=5), did not complete three-month follow-up (n=118), missing DJCS data (n=9), missing covariate data (n=3); [b] standard deviation; [c] independent samples t-test; [d] Chi-square test; [e] 12-Item General Health Questionnaire; [f] interquartile range; [g] Mann-Whitney U test

Appendix 3. Participant characteristics, total ambulance and ED counts, and results of adjusted generalised linear models for ambulance and emergency department contacts, reported as adjusted incidence rate ratios (AIRR) among a cohort of men who regularly injected drugs prior to imprisonment who were released from prison between September 2014 and May 2016 in Victoria, Australia (N=265).

	**Participants (N=265)**	**1. Ambulance Contacts (n=77)**			**2. Emergency Department Contacts (n=123)**		
	n (%)	count	AIRR (95%CI)	p-value	count	AIRR (95%CI)	p-value
Age at baseline (mean, [SD [a]])	36 (8)	-	1.02 (0.99–1.05)	0.295	-	0.96 (0.93–0.99)	0.016
Aboriginal and/or Torres Strait Islander	43 (16)	7	0.76 (0.30–1.89)	0.554	10	0.51 (0.22–1.15)	0.106
Historical psychiatric admission	84 (32)	37	1.39 (0.74–2.58)	0.304	69	1.90 (1.15–3.14)	0.012
Post-release OAT [b]							
None	156 (59)	51	1.00		81	1.00	
Interrupted	34 (13)	11	0.54 (0.21–1.34)	0.182	14	0.54 (0.23–1.24)	0.144
Complete	75 (28)	15	0.33 (0.14–0.76)	0.009	28	0.43 (0.22–0.83)	0.012
Self-reported fair or poor health [c]	123	26	1.41 (0.71–2.77)	0.326	36	1.07 (0.58–1.94)	0.836
Times moved since index release [d]							
0	113 (43)	21			37		
1–2	78 (29)	22	1.47 (1.04–2.07)	0.029	40	1.22 (0.91–1.65)	0.182
3+	74 (28)	34			46		
GP [e] consultations (median, [IQR [f]])	3 (1–6)	-	1.07 (1.03–1.12)	0.001	-	1.06 (1.02–1.10)	0.004
C-GHQ-12 score (median, [IQR [e]])	3 (1–6)	-	0.90 (0.81–0.99)	0.038	-	0.96 (0.88–1.04)	0.318
Any IDU [g] since baseline interview	221 (83)	66	1.29 (0.57–2.90)	0.543	107	1.26 (0.63–2.54)	0.516
Constant	-	-	0.00 (0.00–0.00)	<0.001	-	0.01 (0.00–0.04)	<0.001

[a] Standard deviation; [b] opioid agonist treatment; [c] versus good, very good, or excellent health; [d] categories modelled as a continuous function; [e] general practitioner; [f] interquartile range; [g] injecting drug use.
